# Dataset on nurses' knowledge, attitude and practice in pressure injury prevention at Sabah, Malaysia

**DOI:** 10.1016/j.dib.2023.109193

**Published:** 2023-04-28

**Authors:** Deena Clare Thomas, Rose A. Nain

**Affiliations:** Department of Nursing, Faculty of Medicine and Health Sciences, Universiti Malaysia Sabah, UMS Road, 88400, Kota Kinabalu, Sabah, Malaysia

**Keywords:** Pressure ulcer, Care bundle, Survey, Questionnaire, Nursing, Pieper-Zulkowski, KAP

## Abstract

Pressure injuries are a significant problem in healthcare, and understanding the knowledge and practices of nurses in this area is critical to improving patient outcomes. This article presents dataset concerning a survey conducted to assess the knowledge, attitudes, and practices of pressure injury prevention and care among nurses in public hospitals in the West Coast division of Sabah, Malaysia. The study involved 448 nurses who completed a structured questionnaire between April and December 2021, using the Malay version of the Pieper-Zulkowski-Pressure Ulcer Knowledge Test (PZ-PUKT) 2016 questionnaire. The questionnaire included socio-demographic information and three outcome measures related to pressure injury prevention. Quantitative descriptive statistical analysis was used to analyze the survey results. The data suggest that this survey provides insights into nurses' knowledge, attitudes, and practices regarding pressure injury prevention and could inform the development of interventions to improve the prevention and management of pressure injuries in public hospitals.

**Specifications Table**

**Table:** 

Subject	Nursing and Health Professions
Specific subject area	Nurses' KAP in pressure injury prevention
Type of data	Table
How data were acquired	Data were collected via an online questionnaire
Data format	Raw data and analysed
Description of data collection	The name of the file containing the relevant information for our study is 'KAP DATASET 07042023.xlsx'. The dataset displays the responses of 448 nurses with a minimum of three months of work experience in public hospitals that provide pressure injury care. The data were obtained through a questionnaire administered prior to continuous nursing education series related to pressure injury care which was conducted by the researcher team. An invitation to participate in this study was sent through the nurse manager in each hospital. A link was given to each participant, and they were required to enroll using a unique ID given by the researcher. This dataset includes responses to a survey administered to study participants. This questionnaire aimed to assess participants' knowledge, attitudes, and practices (KAP) related to the care and prevention of pressure injuries. The questions within the dataset have been coded with alphanumeric codes that correspond to their position within the survey. Responses were coded using numerical values or text that reflected each participant's answer. The knowledge section utilised response codes as either correct or incorrect. Correct responses received a score of 1, while incorrect ones received zero marks. These scores were then aggregated and presented as percentage and level categories to assess the participants' knowledge. For the attitude and practice questionnaires, Likert scale responses were coded using string values; each response option received its own score, which could then be summed up and presented in percentage and level categories for data analysis and presentation.
Data source location in	Data was stored at: Institution: Universiti Malaysia Sabah City/Town/Region: Kota Kinabalu/Sabah Country: Malaysia
Data accessibility	Repository name: Mendeley data Data identification number: DOI:10.17632/gmmyws36h3.5 Direct URL to data: https://data.mendeley.com/datasets/gmmyws36h3/5

## Value of the Data

• The dataset can serve as a valuable baseline for relevant stakeholders to plan continuous nursing education that aims to improve the quality of nursing care, particularly related to pressure injury prevention among hospitalised patients.
• The data highlights the importance of identifying nurses' knowledge, attitudes, and practices regarding pressure injury care and prevention, as this serves as the foundation for successful prevention measures for pressure injuries.
• This dataset is highly relevant to researchers interested in conducting intervention studies and systematic literature reviews focused on improving knowledge, attitudes, and practices related to pressure injury prevention.
• The dataset provides a rich information source on the challenges that nurses face in implementing pressure injury prevention practices in a real-world clinical setting. Therefore, it can further justify developing targeted interventions to address these challenges and improve patient outcomes.
• The data offers a unique perspective on the factors that contribute to successful pressure injury care and prevention, including the role of organisational culture, team dynamics, and the availability of resources. This information can help healthcare organisations and policymakers design and implement effective strategies to reduce the incidence of pressure injuries and improve the quality of care for hospitalised patients.

## 1 Objective

Pressure injuries are a significant problem in healthcare, and nurses play a crucial role in preventing and managing them. It is essential for nurses to have a good understanding of pressure injury care because this condition can lead to serious complications, such as infections and sepsis, and can increase hospital length of stay and healthcare costs. This survey aimed to evaluate the knowledge, attitudes, and practices of pressure injury care and prevention among nurses in public hospitals. The dataset will add value to much research on pressure injury care and prevention knowledge and determining the targeted interventions to address these challenges and improve patient outcomes.

## 2 Data Description

The dataset in the CSV format and translated questionnaire Pieper-Zulkowski-Pressure Ulcer Knowledge Test (PZ-PUKT) is available at Mendeley Data at https://data.mendeley.com/datasets/gmmyws36h3/5 [1]. The name of the file containing the dataset for our study is ``KAP DATASET 07042023.xlsx''. This dataset includes responses to a questionnaire that was administered to participants. The questionnaire was designed to assess participants' knowledge, attitudes, and practices related to the care and prevention of pressure injuries. The questionnaire in the dataset is “KAP English”. The data set also includes a file named “KAP code book” which explains the coded questionnaire. The questions in the dataset were coded using a combination of numerical and alphanumeric codes. Each question was identified by a unique code that corresponds to its position in the questionnaire. The responses to each question were coded using numerical values or text indicating the participant's answer. For the knowledge section of our study, responses were coded using a binary string value indicating correct or incorrect answers. Correct answers were given a score of 1 mark, while incorrect answers received 0 marks. These scores were then summed up and presented as a percentage and level categories to assess participants' knowledge. The Likert scale was coded using string values for the attitude and practice questionnaire, but each response option was assigned a corresponding score. Similar to the knowledge questionnaire, the score was summed up and presented as a percentage and level categories to facilitate analysis and interpretation of the dataset.

The dataset contains the raw and analysed data of 448 nurses' socio-demographic characteristics who had been working in public hospitals providing pressure injury care for at least three months. The inclusion criteria were identified by the nurse managers in each respective hospital. The data also contain nurses' knowledge of pressure injury using the 28-items of Pieper-Zulkowski-Pressure Ulcer Knowledge Test (PZ-PUKT), nurses' attitudes and practice related to pressure injury. Table 1 shows the socio-demographic characteristics of participants of 448 nurses. Table 2 presents the nurses' knowledge regarding pressure injury care and prevention, along with the number of correct responses for each of the 28 items in the questionnaire. Table 3 displays nurses' attitudes towards pressure injury prevention, while Table 4 shows the nurses' practice scale in pressure injury prevention. Both data were presented through the distribution of Likert scale responses. Table 5 summarises the scores for knowledge, attitude, and practice related to pressure injury care and prevention, as well as their corresponding level categories.

**Table 1:** Socio-demographic characteristics of participants (*N* = 448).

Variables	n (%)	Variables	n (%)
**Demographic**		**Ward/Unit**	
**Gender**		Medical	159 (35.2)
Male	20 (4.5)	Surgical	102 (22.3)
Female	428 (95.5)	Rehabilitation	127 (28.1)
		orthopedic	63 (14.3)
			
**Age, *M(SD*)**	*33.49 (6.53)*	**Aware of NPUAP/EPUAP International PI**	
**Age categories**		**Prevention and Treatment Guidelines?**	
20 – 29 years	126 (28.1)	Yes	130 (29.0)
30 – 39 years	254 (56.7)	No	318 (71.0)
≥ 40 years	68 (15.2)		
			
**Ethnicity**		**Level of Education**	
Sabahan Natives	359 (80.1)	Diploma	366 (81.7)
Sarawakian Natives	10 (2.2)	Degree	66 (14.7)
Malay	54 (12.1)	Advanced Dip / Certificate	6 (1.3)
Chinese	8 (1.8)	Master / PhD	10 (2.2)
Indian	2 (0.5)		
Others	15 (3.3)		
			
**Working experience category**		**Advanced Wound Course**	
< 1 year	22 (4.9)	Yes	12 (2.7)
1 – 4 years	80 (17.9)	No	436 (97.3)
5 – 10 years	210 (46.9)		
≥ 10 years	136 (30.4)		
			
**Frequency handling PI cases/week**			
Never	52 (11.6)		
Almost never	64 (14.3)		
Sometime/Occasionally	220 (49.1)		
Almost every time	102 (22.8)		
Every time	10 (2.2)

**Table 2:** Nurses' Knowledge toward Pressure Injury Care and Prevention (*N* = 448).

No.	Items	Frequency, n (Percentage, %)	
		Correct	Incorrect
1.	Hot water and soap may dry the skin and increase the risk for pressure ulcers.	298 (66.5)	150 (33.5)
2.	Chair-bound persons should be fitted for a chair cushion.	386 (86.2)	62 (13.8)
3.	A person confined to bed should be repositioned based on individual's risk factors and the support surface's characteristics.	428 (95.5)	20 (4.5)
4.	A pressure injury/ulcer scar will break down faster than unwounded skin.	382 (85.3)	66 (14.7)
5.	The goal of palliative care is wound healing.	154 (34.4)	294 (65.6)
6.	Dragging the patient up in bed increases friction.	382 (85.3)	66 (14.7)
7.	Small position changes may need to be used for patients who cannot tolerate major shifts in body positioning.	386 (86.2)	62 (13.8)
8.	An incontinence patient should have a toileting care plan.	424 (94.6)	24 (5.4)
9.	A pressure redistribution surface manages tissue load and the climate against the skin.	340 (75.9)	108 (24.1)
10.	When possible, high-protein oral nutritional supplements should be used in addition to the usual diet for patients at high risk for pressure injury/ulcers.	414 (92.4)	34 (7.6)
11.	The home care setting has unique considerations for support surface selection.	352 (78.6)	96 (21.4)
12.	Donut devices/ring cushions help to prevent pressure injury/ulcers.	72 (16.1)	376 (83.9)
13.	A specialty bed should be used on areas at risk for shear injury.	80 (17.9)	368 (82.1)
14.	Persons at risk for pressure injury/ulcers should be nutritionally assessed (i.e., weight, nutrition intake, blood work).	420 (93.8)	28 (6.3)
15.	Critical care patients may need slow, gradual turning because of being hemodynamically unstable.	368 (82.1)	80 (17.9)
16.	Staff education alone may reduce the incidence of pressure injury/ulcers.	192 (42.9)	256 (57.1)
17.	A footstool/footrest should not be used for an immobile patient whose feet do not reach the floor.	148 (33.0)	300 (67.0)
18.	Massage of bony prominences is essential for quality skin care.	108 (24.1)	340 (75.9)
19.	Poor posture in a wheelchair may be the cause of a pressure injury/ulcer.	312 (69.6)	136 (30.4)
20.	For persons who have incontinence, skin cleaning should occur at the time of soiling and at routine intervals.	418 (93.3)	30 (6.7)
21.	Patients who are spinal cord injured need knowledge about pressure injury/ulcer prevention and self-care.	424 (94.6)	24 (5.4)
22.	Persons, who are immobile and can be taught, should shift their weight every 30 min while sitting in a chair.	110 (24.6)	338 (75.4)
23.	Selection of a support surface should only consider the person's level of pressure injury/ulcer risk.	98 (21.9)	350 (78.1)
24.	It is not necessary to have the patient with a spinal cord injury evaluated for seating.	176 (39.3)	272 (60.7)
25.	To help prevent pressure injury/ulcer, the head of the bed should be elevated at a 45-degree angle or higher.	200 (44.6)	248 (55.4)
26.	Urinary catheter tubing should be positioned under the leg.	270 (60.3)	178 (39.7)
27.	Pressure injury/ulcer may be avoided in patients who are obese with the use of properly sized equipment.	362 (80.8)	86 (19.2)
28.	Pressure injury/ulcers are a lifelong concern for a person who is spinal cord injured.	428 (95.5)	20 (4.5)

**Table 3:** Nurses' attitude in pressure injury care and prevention (*n* = 448).

No.	Items	Frequency, *n* (Percentage, %)				
		Strongly disagree	Disagree	Mixed feeling	Agree	Strongly agree
1.	In your view are all patients at potential risk of developing pressure injury (PI)?	10 (2.2)	116 (25.9)	34 (7.6)	214 (47.8)	74 (16.5)
2.	Do you think pressure injury prevention is time consuming to carry out?	26 (5.8)	96 (21.4)	50 (11.2)	200 (44.6)	76 (17.0)
3.	Do you have willingness to care for patients with pressure injury?	8 (1.8)	8 (1.8)	30 (6.7)	288 (64.3)	114 (25.4)
4.	Do you feel that priority of care is given for patients who are at risk of pressure injury?	12 (2.7)	8 (1.8)	32 (7.1)	266 (59.4)	130 (29.0)
5.	Do you believe that most pressure injury can be prevented?	10 (2.2)	14 (3.1)	26 (5.8)	210 (46.9)	188 (42.0)
6.	Do you think patients who are admitted receive adequate prevention of pressure injury while in bed seated?	10 (2.2)	66 (14.7)	118 (26.3)	224 (50.0)	30 (6.7)
7.	Do you think pressure injury risk assessment should be regularly carried out on all patients during their stay in hospital?	10 (2.2)	12 (2.7)	14 (3.1)	226 (50.4)	186 (41.5)
8.	Do you perceive that nurses hold major responsibilities when patients are vulnerable to pressure injury?	12 (2.7)	46 (10.3)	54 (12.1)	222 (49.6)	114 (25.4)

**Table 4:** Nurses' practice on pressure injury care and prevention (*N* = 448).

No.	Items	Frequency, *n* (Percentage, %)				
		Never	Rarely	Sometimes	Often	Always
1.	I observe how other nurses assess the risk factors	6 (1.3)	14 (3.1)	82 (18.3)	164 (36.6)	182 (40.6)
2.	I identify common contributing factor of pressure injury/ulcer	4 (9.0)	16 (3.6)	60 (13.4)	170 (37.9)	198 (44.2)
3.	I do a skin assessment	4 (9.0)	10 (2.2)	38 (8.5)	114 (25.4)	282 (62.9)
4.	I use risk assessment scale	26 (5.8)	28 (6.3)	34 (7.6)	78 (17.4)	282 (62.9)
5.	I document all data	14 (3.1)	14 (3.1)	20 (4.5)	106 (23.7)	294 (65.6)
6.	I assess and provide management of pain	12 (2.7)	22 (4.9)	36 (8.0)	102 (22.8)	276 (61.6)
7.	I perform skin care as a routine work	10 (2.2)	16 (3.6)	34 (7.6)	98 (21.9)	290 (64.7)
8.	I place the pillow under the patient's leg	10 (2.2)	18 (4.0)	94 (21.0)	168 (37.5)	158 (35.3)
9.	I use water filled glove under the patient's leg	60 (13.4)	62 (13.8)	132 (29.5)	120 (26.8)	74 (16.5)
10.	I use or advice caregiver to use creams or oils	30 (6.7)	20 (4.5)	66 (14.7)	144 (32.1)	188 (42.0)
11.	I pay more attention to pressure points	6 (1.3)	8 (1.8)	24 (5.4)	112 (25.0)	298 (66.5)
12.	I perform lab test	102 (22.8)	78 (17.4)	112 (25.0)	90 (20.1)	66 (14.7)
13.	I provide vitamin and food	28 (6.3)	20 (4.5)	70 (15.6)	158 (35.3)	172 (38.4)
14.	I monitor a protein and calorie diet	20 (4.5)	24 (5.4)	68 (15.2)	180 (40.2)	156 (34.8)
15.	I avoid dragging	256 (57.1)	106 (23.7)	56 (12.5)	12 (2.7)	18 (4.0)
16.	I always use a special mattress	16 (3.6)	30 (6.7)	94 (21.0)	118 (26.3)	190 (42.4)
17.	I avoid massage at the bony prominence area and patient's back	86 (19.2)	92 (20.5)	136 (30.4)	62 (13.8)	72 (16.1)
18.	I avoid using donut – shape (ring) cushion	56 (12.5)	60 (13.4)	116 (25.9)	102 (22.8)	114 (25.4)
19.	I turn a patient position every two hours	12 (2.7)	14 (3.1)	36 (8.0)	132 (29.5)	254 (56.7)
20.	I put pillows under the patient's leg ankle	14 (3.1)	24 (5.4)	68 (15.2)	162 (36.2)	180 (40.2)
21.	I always attend seminars	50 (11.2)	152 (33.9)	156 (34.8)	48 (10.7)	42 (9.4)
22.	I give advice to the patient or caregiver	14 (3.1)	42 (9.4)	72 (16.1)	124 (27.7)	196 (43.8)

## 3 Experimental Design, Materials, and Methods

### 3.1 Participants, Response Rate and Data Collection Procedure

A cross-sectional study was conducted from April to December 2021 to investigate nurses' knowledge, attitudes, and practices related to pressure injury prevention in public hospitals in the West Coast division of Sabah. A total of 620 nurses were identified and invited to participate. Those who agreed were given a unique ID. Of the 620 invited nurses, 448 consented to participate, indicating an acceptable response rate of 72.3% [2]. The sample size was based on similar studies [3], [4], [5], and samples were selected using universal sampling [2].

**Table 5:** Knowledge, attitude and practice score and level categories.

Domains	*Mean (SD)*	Frequency (*n*)	Percentage (%)
**Knowledge score**	64.12 (9.11)		
Low (< 69)		304	67.9
Moderate (70 – 79)		126	28.1
High (≥ 80)		18	4.0
		
**Attitude score**	27.78 (2.89)	238	53.1
Positive		210	46.9
Negative			
		
**Practice score**	82.18 (9.85)		
Good practice (≥ 82.1)		150	54.5
Poor practice (< 82.1)		204	45.5

### 3.2 Data Collection and Instruments

Prior to the study, ethical approval was obtained from the Medical Research Ethics Committee of Malaysian Ministry of Health NMRR-21-146-58331 (IIR). Consent was taken from each participant to ensure that participants' anonymity and confidentiality were maintained. Data was collected using an online self-administered research questionnaire consisting of three sections of the Malay version of the Pieper-Zulkowski-Pressure Ulcer Knowledge Test (PZ-PUKT) version 2016 questionnaire [3]. The knowledge section consisted of 28 items, with participants required to choose between “True,” “False,” and “Do not Know,” and answers were scored as either “correct = 1 score” or “incorrect = 0 score.” Nurses' knowledge levels were categorised as low (< 69), moderate (70–79), or high (≥80). The attitude section consisted of 8 items, in which the participants were required to respond based on a five-point Likert scale ranging from “Strongly Disagree” to “Strongly Agree.” Scores above the mean indicated a positive attitude, whereas scores below the mean were regarded as indicative of a negative attitude [3], [4], [5], [6]. The practice section consisted of 22 items, with a five-point Likert scale from 'never' to 'always'. A score above the mean was considered good practice, while a score below the mean was considered poor practice. The items showed good content validity (I-CVI 0.79 to 1.00 and Kappa between 0.67 to 1.00) and good to excellent measurement with an intraclass correlation coefficient (ICC = 0.78, 95% CI: 0.66–0.83) and Cronbach's α of 0.70 for knowledge, 0.69 for attitude, and 0.89 for practice, indicating good to excellent reliability [7,8].

Descriptive data analysis was performed using SPSS Statistics software for Windows version 26 (IBM Corp., Armonk, NY). The following information was included in the sample.

**Demographic data:** Several socio-demographic questions were asked of the respondents. These include gender, age, age group, ethnicity, working experience, ward name, frequency of handling PI cases/week, awareness of NPUAP/EPUAP International PI Prevention and Treatment Guidelines and attended wound care course.

**Knowledge, Attitude and Practice (Pressure Injury Care and Prevention):** There were 28 knowledge-based questions in the instruments. The total 28 questions mainly refer to the pressure injury prevention items. Eight questions measured these components for the nurses' attitude, while 22 questions measured nurses' practice in pressure injury prevention.

## Ethics Statements

The data collection process adhered to the ethical principles of the Declaration of Helsinki. An online questionnaire was employed to maintain anonymity, and the collected data was coded. The initial page of the questionnaire provided a brief overview of the study's objectives and an online consent form. Participants who opted to participate in the survey were provided access to the questionnaire. Therefore, no participant was subject to coercion during the survey. Ethical approval number: NMRR-21-146-58331 (IIR). Link: https://nmrr.gov.my/research-directory/78256e09-e0bb-45c8-bd98–73e21c3028fc.

## CRediT authorship contribution statement

**Deena Clare Thomas:** Conceptualization, Methodology, Data curation, Investigation, Writing – review & editing. **Rose A. Nain:** Data curation, Writing – original draft, Validation, Writing – review & editing.

## Declaration of Competing Interest

The authors declare that they have no known competing financial interests or personal relationships that could have appeared to influence the work reported in this paper.

## Data Availability

Dataset of Pressure Injury Prevention Knowledge, Attitude and Practice of Nurses at Sabah, Malaysia (Original data) (Mendeley Data).
